# Levels of maternal care in dogs affect adult offspring temperament

**DOI:** 10.1038/srep19253

**Published:** 2016-01-13

**Authors:** Pernilla Foyer, Erik Wilsson, Per Jensen

**Affiliations:** 1 IFM Biology, AVIAN Behaviour Genomics and Physiology group, Linköping University, 581 83 Linköping, Sweden; 2 Department of Military Studies, Military-Technology Division, Swedish Defence University, 115 93 Stockholm, Sweden; 3 Swedish Armed Forces Dog Training Unit, Märsta, Sweden

## Abstract

Dog puppies are born in a state of large neural immaturity; therefore, the nervous system is sensitive to environmental influences early in life. In primates and rodents, early experiences, such as maternal care, have been shown to have profound and lasting effects on the later behaviour and physiology of offspring. We hypothesised that this would also be the case for dogs with important implications for the breeding of working dogs. In the present study, variation in the mother-offspring interactions of German Shepherd dogs within the Swedish breeding program for military working dogs was studied by video recording 22 mothers with their litters during the first three weeks postpartum. The aim was to classify mothers with respect to their level of maternal care and to investigate the effect of this care on pup behaviour in a standardised temperament test carried out at approximately 18 months of age. The results show that females differed consistently in their level of maternal care, which significantly affected the adult behaviour of the offspring, mainly with respect to behaviours classified as Physical and Social Engagement, as well as Aggression. Taking maternal quality into account in breeding programs may therefore improve the process of selecting working dogs.

Early experiences affect the phenotypic, genomic and behavioural traits of the adult animals, as has been shown by a range of studies in different species. For example, Levine *et al.*^1^, showed that previously maternally separated adult rats were less reactive, more explorative and more emotionally stable compared with controls and also exhibited lower plasma corticosterone levels over time. In altricial species, offspring are born in a state of high neural immaturity, and the nervous system rapidly develops via an intense synaptogenesis^2^, during which environmental influences can have a profound and lasting effect on an animal’s behaviour^3^. In mammals, the neonatal period is also a time of significant social interaction, so it is an important time for the development of social behaviour^4^, as well as the stress response^5^.

Early postnatal handling by humans has important long-term consequences in rodents, which have inspired numerous experiments. It is clear that the most important effects of neonatal handling are primarily due to the fact that human handling induces increased maternal care^5^,^6^. For example, Caldji *et al.*^7^, showed that responses to novelty and restraint differed among rat offspring according to the amount of maternal care received, which is measured as low and high levels of licking/grooming and arched-back nursing (LG-ABN). In this study, the adult offspring of low LG-ABN mothers showed an increased startle response, decreased open-field exploratory behaviour and a longer latency for eating food in a novel arena. Cross-fostering studies have also shown that individual differences in fearfulness and maternal care of the offspring can be a function of the behaviour of the rearing mother rather than the biological mother^8^,^9^. It has been proposed that the mother provides primary environmental cues to her young during the early postnatal period, thereby adaptively modifying their behaviour with respect to their predicted future environment^10^. Hence, postpartum experiences can alter neurological development^11^ and be important in the shaping of the physiological and behavioural stress response in rodents^9^.

In other mammalian species such as prairie voles^12^, monkeys^13^,^14^, and sheep^15^, early mother-offspring interactions affect offspring behaviour. If similar effects exist in dogs, they may have considerable consequences for dog breeding. Dogs are bred for various types of work purposes, and the selection programs are mostly based on the evaluation of behaviour post puberty, which is based on the assumption that genetic factors explain most of the variance in adult behaviour^16^. However, heritability studies have revealed only a small contribution of genetics to the phenotypic variation^17^,^18^,^19^, and aspects of the rearing environment have been found to exert clear effects on adult behaviour^20^. Therefore, it is possible that early experiences and variation in maternal care may be at least as important as genetics.

In this study, records and observations of 22 litters of German Shepherd dogs bred to work in the Swedish Armed Forces (SAF) were analysed to evaluate variations in maternal care and their effects on behaviour and temperament in puppies at approximately one and a half years of age. We carried out detailed behaviour recordings during the suckling periods and obtained the adult behavioural traits from a standardised temperament evaluation test (SAF T-test) used by the SAF as an instrument for selecting suitable military working dogs (MWD). The SAF has conducted their German Shepherd breeding program since 2005. To date, approximately 30% have been approved to work as MWDs or within the police force. Most dogs are rejected because their temperament profiles do not fit the needs of the Armed Forces, but some are also rejected for medical reasons. Because of the relative large number of rejected dogs due to temperamental mismatch and a possible link between early experiences and later temperament, we investigated the effects of early maternal care on the behaviour of adult dogs.

The aim of this study was to assess individual differences in maternal care and examine their impact on the behaviour and temperament of the young. We hypothesised that the degree and quality of maternal care would vary between dogs in a consistent manner during the suckling period and that more maternal care would lead to more confident, less reactive and more explorative puppies.

## Methods

Observations of mother-pup interactions were conducted on 22 different litters (a total of 94 pups: 42 males and 52 females) through continuous video recordings for three weeks postpartum. The results from the later evaluation of the offspring using the standardised temperament test (SAF T-test) were retrieved from the SAF Dog Training database.

### Ethical note

All of the experiments performed by us in this study were conducted in accordance with the approved guidelines of the Regional ethical committee for animal experiments in Linköping, Sweden (Permit number: 51–13).

### Subjects

All of the dogs in this study, 22 females and their litters, were from a population of outbred German Shepherds, bred as part of the Swedish Armed Forces selective dog-breeding program between 2011 and 2013. The females were between 2 and 8 years old (parity/number of previous litters; 1–4), and the litter sizes varied from 1 to 10 (average 5 ± 2,3 s.e.m). None of the dogs lived permanently at the kennel situated in Sollefteå, Sweden; the females lived in private foster homes across Sweden and arrived at the kennel approximately three weeks before the estimated whelping day.

For the first week at the kennel, the females were housed separately in 4.5-m^2^ quarantine rooms connected to a 13.5-m^2^ outdoor enclosure. They were then transferred to the 9-m^2^ whelping room, where they were individually housed until the pups were born. All of the whelping rooms had a window that admitted natural light, and direct access to a 9-m^2^ outdoor enclosure. During housing at the kennel facility, the females received food four times a day (07.30, 11.00, 14.00 and 19.20). Water was offered *ad libitum*, and light was automatically turned on at approximately 07.00 in the morning and switched off at approximately 20.00 at night. Walks on leash with a handler were undertaken three times a day, and the rooms were cleaned once a day during the morning walks. Females stayed with their pups until they were weaned at the age of eight weeks. At that time, both females and pups were allocated to separate foster homes. Pups were later brought back at 15–20 months of age for evaluation. Approved dogs were kept for further training, whereas non-approved dogs were sold as companion dogs.

Whelping was monitored via a video link in a nearby room. In the whelping room, there was a 1.0*1.0*0.3-m puppy box equipped with a bar and a detachable front. The flooring in the box was a soft bed (Vetbed) and a linoleum carpet covered the floor of the room. The temperature was kept at 21 degrees Celsius. The front of the whelping box was detached at the end of the third week, and the pups were allowed access to the entire room. At the same time, females received access to twice the space via an elevated interconnection to an identical room next door, and they had access to a wall-mounted elevated shelf. Pups were weighed every week and were vaccinated in accordance with current recommendations. Beginning in the third week, pups were allowed access to solid food, and from six weeks of age, they were allowed daily visits to an enriched area, either indoors or outdoors depending on the season and weather. Pups were also taken for car rides and walks in the woods during their stay at the kennel as a part of their socialisation.

### Recording Procedure of Mother-Pup Interaction

Behaviour was recorded continuously for all litters from birth until three weeks of age with a surveillance camera (Sony SNCRZ25N PTZ with IR-led for night vision), and video files were stored for later scoring and analysis offline. Behavioural recordings were done continuously for every second hour over a 24-h period once per week while the pups were mainly confined to the pup box (before they became mature enough to move around and the front wall of the puppy box was detached), i.e., the first, seventh, 14^th^ and 21^st^ day postpartum. This observation schedule provided 12 h of continuous recordings per observation day for each female and her litter. For technical reasons, the last recording (three-weeks old pups) was performed on day 18 in one case, on day 19 in two cases, and on day 20 in three cases.

The variables recorded were:

*Mother in Box: the time in seconds during which the mother had both of her front legs in the pup box.

*Lying in contact: the time in seconds during which the mother was lying in the pup box with elbows on the ground and in physical contact (tail and limbs excluded) with at least one pup.

*Nursing: Duration in seconds of nursing bouts with at least one pup lined up at the udder.

*Licking: Duration in seconds spent licking pups.

*Sniff/Poke: Frequency of sniffing, poking or moving a pup around with her nose.

All variables except for Mother in Box were divided by litter size, so the actual measurements analysed were the durations and frequencies per pup in each litter.

Videos were decoded by four different people with the largest proportion (16/22) of videos decoded by one person (first author). Inter-observer reliability was determined by 3 × 20-min of video samples for each observer (i.e., all observers re-coded the same samples). Inter-observer reliability between the main observer and the others was 89% on average, ranging from 100% (max) for *Mother in box* and *Lying in contact*, to 65% (min) for *Licking.*

Because it was not possible to reliably distinguish each individual pup in the videos, the litter was treated as the observation unit. In later analyses, every member of a litter was therefore assigned the same scores for the maternal care received.

### Temperament Test

At 15–18 months of age, all dogs reared by the mothers included in the study were called in from their foster homes and subjected to the standardised temperament test used by the Swedish Armed Forces (SAF T-test) in the selection process for prospective MWDs. The tests were carried out at five different sites: Ronneby, Säve, Märsta, Solleftå and Luleå, and the dogs were brought to the test site by their foster families in private cars a few hours before the start of the test.

Details of the test methods are described in Wilsson and Sinn^21^. Briefly, the SAF T-test consists of 12 different sub-tests, which evaluate the dogs’ reactions to a range of situations, from social interactions and co-operation with humans to potentially frightening stimuli such as loud noises or suddenly appearing dummies. The dogs were assessed using a Behavioural Rating (BR) protocol including 25 behavioural variables. Each variable was scored by the same experienced test leader for all dogs on a scale ranging from 1–5 (see Wilsson and Sinn (2012) for details about scoring). During the test, a handler, usually a member of the foster family accompanied the dogs and was guided by a test leader.

### Statistical analysis

#### Differences in Maternal Care

For the behavioural recordings of the mother-pup interactions (MPI), a principal component analysis (PCA) was performed on the five variables from the ethogram. We first performed a PCA for the aggregated values for each mother, i.e., the sum of each variable during the four observation days (referred to as Total MPI). Secondly, separate PCAs were done for each of the four sampling days (termed MPI1-MPI4), and sampling adequacy was tested. The correlation matrix for the behavioural recordings was considered appropriate for PCA in all cases (Total MPI; Bartlett´s sphericity χ2 (10) = 133.1, P < 0.001; KMO = 0.760, MPI1; Bartlett´s sphericity χ2 (10) = 109.7, P < 0.001; KMO = 0.771, MPI2; Bartlett´s sphericity χ2 (10) = 102.5, P < 0.001; KMO = 0.578, MPI3; Bartlett´s sphericity χ2 (10) = 103.1, P < 0.001; KMO = 0.685, and MPI4; Bartlett´s sphericity χ2 (10) = 97.9, P < 0.001; KMO = 0.780). Based on the PCAs, PC-scores, which are referred to as MPI-scores, were assigned to each mother.

To examine the individual consistency in MPI-scores between the four different measurement days, Pearson correlation coefficients were calculated for MPI1-MPI4. To examine the individual differences in maternal care, the mothers were divided into two equally sized groups according to their Total MPI score (high and low Total MPI scores), and the means and standard deviations of the five different behavioural variables described above were calculated separately for each group and observation day. Independent samples t-tests were used to calculate the differences in the maternal behaviour variables between the groups.

To investigate which factors that may have affected the Total MPI score, generalized linear models were used. The Total MPI score was used as the response variable, and Parity, Sex-ratio, Litter Size and Trimester of Birth (January-March, April-June, July-September, and October-December) were used as predictors. Significant effects were only observed for Litter size, so the other variables were dropped from the model in subsequent analyses. The probability distribution used was “Normal”, and the link function was “Identity”; significance levels were determined with a Wald chi-squared test with adequate degrees of freedom. An omnibus (likelihood ratio chi-square) test was used to determine the performance of the model versus the intercept, and this was deemed acceptable when the significance level was below 0.05.

#### Effects of Maternal Care on Offspring Temperament

Principal component analysis (PCA) was used to reduce 25 behaviour variables in the standardised temperament test, and the primary factor solution was rotated using the Oblimin with Kaiser normalisation method. Sampling adequacy was tested, and the correlation matrix for the behaviour recordings was considered appropriate for PCA (Bartlett´s sphericity χ^2^_(300)_ = 920.9, P < 0.001; KMO = 0.697).

For the statistical analysis of the relationship between Total MPI score and the behavioural responses in the SAF T-test, we used generalized linear models and the average factor scores of the members in a litter from each of the extracted factors from the SAF T-test as response variables. Hence, the litter was considered to be the statistical unit. As predictors, we used Parity, Sex-ratio, Litter Size, Trimester of Birth and the Total MPI score. Starting with the least significant values, predictors were removed one by one until only significant predictors were left in the model. Because there was only a significant effect of parity for PC2, this predictor was only kept for PC2. The probability distribution used was “Normal”, and the link function was “Identity”. Significance levels were determined with the Wald chi-squared test with adequate degrees of freedom, and an omnibus (likelihood ratio chi-squared) test was used to compare the performance of the model versus the intercept. Performance was deemed acceptable when the significance level was below 0.05, which was the case for all of the results reported here. Results are reported as estimated marginal means with their standard errors. All data were analysed using SPSS 22.0.

## Results

### Differences in Maternal Care

The principal component analysis of the behavioural maternal care data revealed just one factor with eigenvalues greater than one, which explained 80.0% of the variance (N = 22) (Table 1). The factor score was termed Total Mother-Pup Interaction (Total MPI).

**Figure 1:**
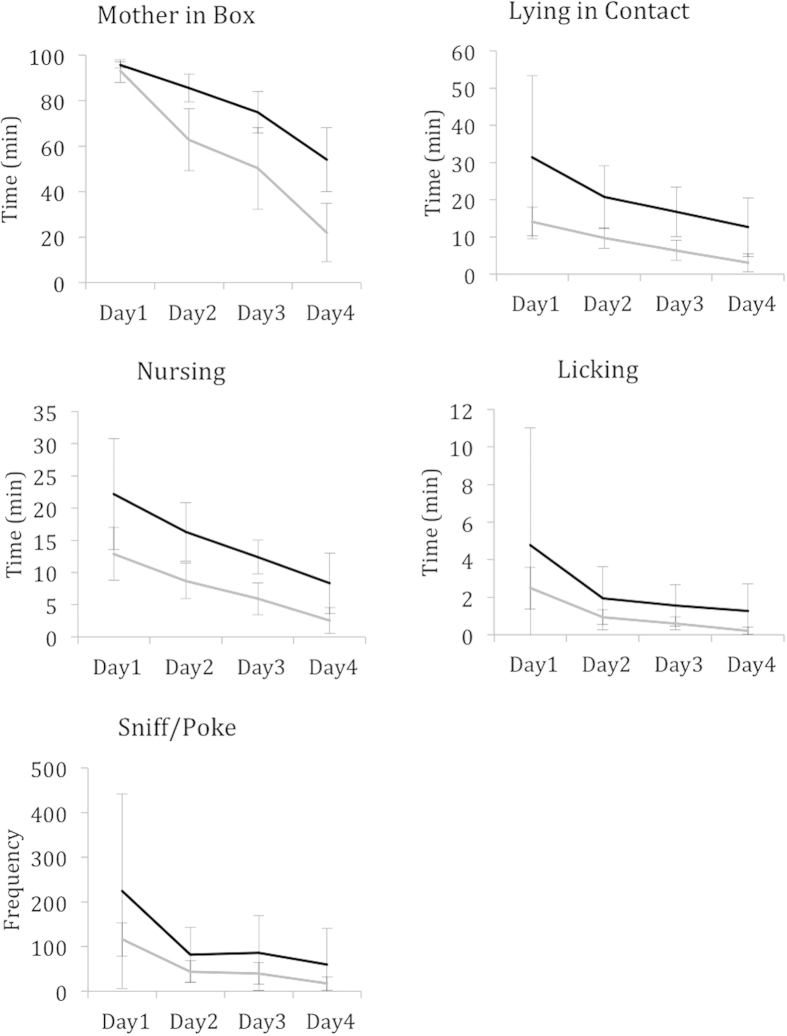
Difference in maternal behaviour between two groups of females. Maternal behaviours during four days of observation over the three first weeks postpartum for five recorded variables: Mother in Box, Lying in contact, Nursing, Licking, and Sniff/poke. Graphs show the mean values (±s.e.m.) for the two groups of females based on their Total MPI scores; N_(high)_ = 11, and N_(low)_ = 11.

The individual females were highly individually consistent in their maternal behaviour over time, and the Pearson correlation for the MPI1-MPI4 scores for the different sampling days ranged from 0.841–0.965 (N = 22, P < 0.001). Furthermore, as measured by their Total MPI score, the females clearly differed from each other in the amount of maternal behaviour they performed. When dividing the mothers into two groups based on their Total MPI score, there was a clear difference between the high and low MPI groups with regard to all five different variables, and all maternal behaviours declined steadily over the four days of recording (Fig. 1). However, the differences on day one were not significant for any variable, and all of the females spent most of their time with their litters on the first day post partum.

Mothers in the high MPI score group showed significantly more maternal behaviour than the low score group for *Mother in Box* (37.0 ± 2.4 vs. 27.6 ± 4.3, t_(20)_ = 6.26, P < 0.000), *Lying in Contact* (9.5 ± 5.3 vs. 3.7 ± 1.2, t_(20)_ = 3.53, P = 0.005) and *Nursing* (6.9 ± 2.2 vs. 3.4 ± 1.1, t_(20)_ = 4.80, P < 0.000). *Licking* and *Sniff/Poke* did not differ significantly between the high and low MPI litters (1.0 ± 1.2 vs. 0.3 ± 0.1, t_(20)_ = 1.89, P = 0.073, and 451.0 ± 431 vs. 217 ± 93, t_(20)_ = 1.76, P = 0.094, respectively).

Total MPI score was significantly affected by Litter size (Wald’s X^2^(1) = 10.7; P = 0.001). Females with small litters (N = 1–5 pups/litter) had higher Total MPI scores than large litters (N = 6–10 pups/litter): 0.51 ± 0.23 vs. −0.61 ± 0.25, respectively.

### Effects of Maternal Care on Offspring Temperament

The principal component analysis of the behavioural data from the SAF T-test revealed eight factors with eigenvalues greater than one, which together explained 73.6% of the variance (N = 76). Based on the coherence with the results from earlier studies of similar datasets^20^,^21^,^22^, and a PC loading of 0.5 or higher as an arbitrary criterion for a variable to be considered relevant to the interpretation of a specific PC, we decided to consider the first four factors and name them according to previous studies; Confidence, Physical Engagement, Social Engagement and Aggression (Table 2). Together, the four factors explained 53.1% of the variance.

**Figure 2:**
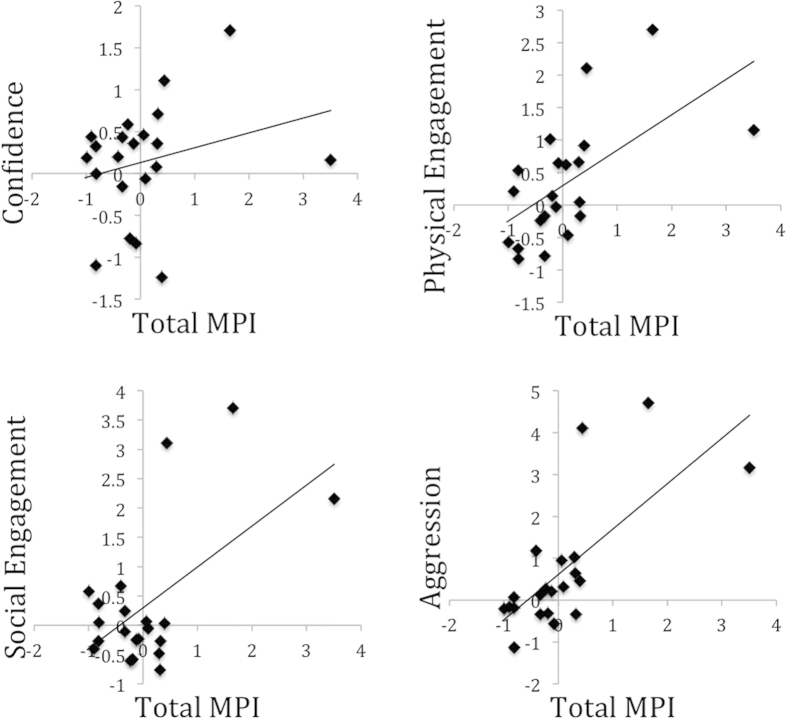
The relationship between Total MPI score and Confidence, Physical Engagement, Social Engagement and Aggression. Scatter plots of litter mean PC scores for Confidence, Physical Engagement, Social Engagement and Aggression plotted against the Total MPI scores; N = 22.

There was a significant relationship between Total MPI score and Physical Engagement, Social Engagement and Aggression, but no significant association was found between Total MPI score and Confidence in the offspring (Physical Engagement: Wald’s X^2^(1) = 11.5; P = 0.001; Social Engagement: Wald’s X^2^(1) = 10.7; P = 0.001; Aggression: Wald’s X^2^(1) = 21.8; P < 0.001; Confidence: Wald’s X^2^(1) = 1.4; P = 0.229) (Fig. 2).

Apart from the effect of Total MPI score, Social Engagement was also significantly affected by Litter size and Trimester of Birth (Litter size: Wald’s X^2^(1) = 6.0, P = 0.014; Trimester of Birth: Wald’s X^2^(3) = 11.3, P = 0.010). Small litters (Number of pups = 1–5; N = 12) scored higher on average than large litters (Number of pups = 6–10; N = 9) (0.47 ± 0.15 vs. −0.16 ± 0.21, respectively), and litters born during the summer scored higher than litters born during the winter: Apr-Jun (N = 3): 0.29 ± 0.31; Jul-Sep (N = 3): 0.84 ± 0.33; Oct-Dec (N = 6): −0.43 ± 0.21; and Jan-Mar (N = 9): −0.08 ± 0.18. Furthermore, there was a significant relationship between Physical Engagement and Trimester of Birth (Wald’s X^2^(3) = 10.1, P = 0.018), and litters born during the summer months scored higher than litters born during the winter: Apr-Jun (N = 3): 0.30 ± 0.37; Jul-Sep (N = 3): 0.99 ± 0.37; Oct-Dec (N = 6): −0.41 ± 0.26; and Jan-Mar (N = 9): −0.04 ± 0.21. Aggression was also significantly affected by Trimester of Birth (Wald’s X^2^(3) = 15.0, P = 0.002), and again, litters born during the summer months scored higher than litters born during the winter: Apr-Jun (N = 3): 0.19 ± 0.30; Jul-Sep (N = 3): 1.03 ± 0.30; Oct-Dec (N = 6): −0.37 ± 0.21; and Jan-Mar (N = 9): −0.05 ± 0.17.

## Discussion

Our results demonstrate that female German Shepherds varied in a consistent manner in the amount of maternal behaviour provided during the first three weeks postpartum, and this affected both Social and Physical Engagements, as well as Aggression, in the offspring when they became adults. These results clearly demonstrate that maternal care early in the life of dogs affects the behaviour and temperament of the offspring for an extended period in life. This may be of profound importance to the understanding of individual variation in behaviour in dogs and may help to improve breeding schemes for working dogs.

Similar to other altricial mammalian species, maternal behaviour in dogs during the neonatal period primarily consists of feeding the pups and keeping them warm, clean and protected^4^,^23^,^24^. Unfortunately, it was not possible to consistently distinguish between individual pups on our video recordings, so we assigned the same value of maternal care received to all of the individuals in a particular litter. Champagne *et al.*^10^, reported no differences in the amount of licking/grooming received between individual pups within a litter, and studies of dogs have only reported small intra-litter variation in maternal behaviour^25^. Hence, we assume that within-litter variation in maternal care was sufficiently low to allow us to use the litter means.

All five of the maternal behaviours recorded loaded strongly on one single principal component, and there was a high correlation between the MPI values scored separately on the four different observation days. Hence, there is strong justification for assigning a single mother-pup interaction score (MPI) to each female and litter.

Maternal behaviour was highly consistent, and although it steadily declined over time, individual differences remained stable. These results are consistent with previous studies of rats^10^,^26^ and dogs^27^, which indicates that different females in our study may have adopted different mothering styles, which is similar to what is known in rodents^5^,^8^,^9^,^28^,^29^. The Total MPI score should therefore reliably reflect the mothering style of a particular female.

Similar to what was previously reported by Priestnall^30^ for mice, the MPI score was significantly affected by litter size. In a small litter, the possibility of physical contact between the mother and any sibling is greater, which may explain our results. However, it could also be that the larger litters offer each sibling greater opportunities for physical comfort from other pups, which could therefore provide some comfort and decrease the need for maternal care. In this study, we are not able to distinguish between those possibilities, since that would require individual recordings from all pups.

We found no effect of Parity, Sex-ratio or Trimester of Birth on MPI score in this study, although all three have previously been found to affect behaviour^20^. However, the number of animals included here was considerably smaller than that included in previous studies, which could have made it more difficult to detect the effects.

In line with earlier behavioural studies of rodents^7^,^9^,^29^,^31^, variation in maternal care had a clear effect on the behaviour of adult dog offspring. The High Total MPI score significantly affected Physical Engagement, which is similar to results observed in mice^7^. There was also a significant relationship between MPI score and Social Engagement, in which more maternal care was related to more engagement in social activities with humans, again mirroring results in mice Branchi *et al.*^32^. Finally, there was also a significant relationship between Aggression and the MPI score.

Furthermore, the temperament was affected by litter size and season of birth. One possible interpretation of this would be that it could be possible to affect adult behaviour of dogs by adjusting litter sizes and birth times, but the present data do not allow this conclusion. The main reason is that we cannot rule out the possibility that the effects demonstrated are confounded by genetic factors: possibly, dog mothers with a genetic predisposition for high maternal care could at the same time be more prone to have small litters and also to have offspring with a certain temperament profile. Hence, more research is needed on the genetic aspects of maternal behaviour before we can draw any practical conclusions with respect to dog breeding.

These results clearly show that the level of maternal care has a profound effect on behavioural development in dogs. Hence, behavioural variation in dogs can be explained to some extent by variations in the maternal styles of the females. Therefore, when breeding working dogs, it could be important to include evaluations of the mothers used in the selection programs. Our results demonstrate that some of the variation in maternal behaviour can be explained by environmental factors (litter size, time of year), but it is very likely that a large portion is due to genetic differences between females.

## Conclusions

The maternal style of female German Shepherd varied consistently during the first three weeks postpartum. This difference in maternal care affected the behaviour and temperament of the offspring later in life; pups from litters raised by mothers that provided more maternal care scored higher for social engagement, physical engagement and aggression than those brought up by less attentive mothers. The pups’ adult score for confidence was unaffected by maternal style.

## Additional Information

**How to cite this article**: Foyer, P. *et al.* Levels of maternal care in dogs affect adult offspring temperament. *Sci. Rep.* **6**, 19253; doi: 10.1038/srep19253 (2016).

## Figures and Tables

**Table 1:** Results of the principal component analysis of the maternal care behavioural data revealed just one factor with eigenvalues greater than one.

Behavioural Variable	Total Mother-Pup Interaction
Female in Box	0.70
Lying in contact/pup	0.98
Nursing/pup	0.94
Licking/pup	0.90
Sniff/Poke/pup	0.92
Total Variance Explained (%)	80.0

The total variance explained by this factor, termed Total Mother-Pup Interaction (Total MPI), is 80.0%; N = 22.

**Table 2:** Results from the principal component analysis after Oblimin rotation with Kaiser normalisation.

Behavioural Variable	Confidence	Physical Engagement	Social Engagement	Aggression
V. Flight distance	**0.80**	−0.12	−0.04	0.04
V. Secondary response	**0.82**	0.00	−0.12	−0.14
V. Lasting effect	**0.78**	−0.01	−0.18	0.14
G. Fearfulness	**0.65**	0.07	−0.17	**0.64**
G. Secondary response	**0.57**	0.16	−0.19	0.35
G. Lasting effect	**0.66**	0.26	−0.32	0.23
Metal stair	−0.13	**0.50**	0.05	0.42
Reaction on table	−0.06	**0.75**	−0.08	−0.03
Object	0.01	**0.80**	−0.13	−0.07
Affability	0.11	0.17	−**0.86**	0.10
Handling	0.05	0.07	−**0.86**	0.20
Leash	0.19	0.25	−**0.83**	0.16
Reaction in dark room	0.17	−0.17	−**0.66**	0.10
V. Aggression	−0.15	0.04	−0.19	**0.77**
G. Aggression	0.41	−0.11	−0.14	**0.70**
Tug-of-war	0.15	0.48	−0.14	−0.03
Chasing	−0.02	0.26	−0.17	−0.06
Interest in object	0.09	0.05	−0.20	0.20
A. Flight distance	0.46	−0.02	0.12	0.11
A. Secondary response	0.18	0.06	0.11	0.10
A. Lasting effect	0.29	0.18	−0.13	−0.09
Search Intensity	0.17	0.28	−0.06	0.01
Search Persistence	0.25	0.28	−0.13	−0.07
Gun-shot Fear	0.03	0.32	−0.36	0.30
Gun-shot Curiosity	−0.04	0.25	0.19	0.12
% Variance explained	21.7	13.4	11.1	6.8

The table shows the SAF T-test variable loadings of the first four of the six components with eigenvalues >1. Loadings above 0.5 are shown in bold. A – acoustic startle sub-test; G – gradual visual startle sub-test; V – visual startle sub-test. The total variance explained by these four factors is 53.1%. N = 76.
